# The Empirical Fluctuation Pattern of *E. coli* Division Control

**DOI:** 10.3389/fmicb.2018.01541

**Published:** 2018-07-30

**Authors:** Jacopo Grilli, Clotilde Cadart, Gabriele Micali, Matteo Osella, Marco Cosentino Lagomarsino

**Affiliations:** ^1^Santa Fe Institute, Santa Fe, NM, United States; ^2^Centre National de la Recherche Scientifique, Institut Curie, PSL Research University, UMR 144, Paris, France; ^3^Institut Pierre-Gilles de Gennes, PSL Research University, Paris, France; ^4^Department of Environmental Microbiology, Eawag, Dübendorf, Switzerland; ^5^Department of Environmental Systems Science, ETH Zurich, Zurich, Switzerland; ^6^Physics Department, University of Turin, Turin, Italy; ^7^Istituto Nazionale di Fisica Nucleare Sezione di Torino, Turin, Italy; ^8^Sorbonne Université, Paris, France; ^9^Centre National de la Recherche Scientifique, UMR 7238, Paris, France; ^10^IFOM, FIRC Institute of Molecular Oncology, Milan, Italy

**Keywords:** linear response theory, single-cell growth and division, fluctuation patterns, control of cell division, models, theoretical, data interpretation, statistical

## Abstract

In physics, it is customary to represent the fluctuations of a stochastic system at steady state in terms of linear response to small random perturbations. Previous work has shown that the same framework describes effectively the trade-off between cell-to-cell variability and correction in the control of cell division of single *E. coli* cells. However, previous analyses were motivated by specific models and limited to a subset of the measured variables. For example, most analyses neglected the role of growth rate variability. Here, we take a comprehensive approach and consider several sets of available data from both microcolonies and microfluidic devices in different growth conditions. We evaluate all the coupling coefficients between the three main measured variables (interdivision times, cell sizes and individual-cell growth rates). The linear-response framework correctly predicts consistency relations between *a priori* independent experimental measurements, which confirms its validity. Additionally, the couplings between the cell-specific growth rate and the other variables are typically non zero. Finally, we use the framework to detect signatures of mechanisms in experimental data involving growth rate fluctuations, finding that (1) noise-generating coupling between size and growth rate is a consequence of inter-generation growth rate correlations and (2) the correlation patterns agree with a near-adder model where the added size has a dependence on the single-cell growth rate. Our findings define relevant constraints that any theoretical description should reproduce, and will help future studies aiming to falsify some of the competing models of the cell cycle existing today in the literature.

## 1. Introduction

Today, dynamically tracked data of many dividing cells offer the possibility to analyze with great precision and detail the decision process leading to cell division (Osella et al., 2017). While such data are starting to be abundant, measurements of many single cells yield a complex tangle of correlation patterns between growth-related variables, which is often difficult to grasp. Consequently, new theoretical and data-analysis tools need to be developed in order to extract from such data the relevant information to understand the control of cell-cycle progression, cell division and their impact on cell proliferation.

Specifically, one can restrict the question to the control of cell size, where some general principles are emerging from empirical data. *E. coli* cells grow with a cell-specific growth rate, they are born with a cell-specific size, and they divide with a cell-specific cell-cycle time. A cell that deviates from average behavior in size or growth rate can correct by a compensatory deviation in doubling time. A “near-adder” control of cell division (Campos et al., 2014; Taheri-Araghi et al., 2015), whereby the differential size extension in a single cell cycle is independent of the initial size of the cell, is an effective principle governing cell division. Additionally, the distributions of interdivision times and cell sizes across conditions (Iyer-Biswas et al., 2014b; Kennard et al., 2016) show a clear link between average values and the variability of these variables from cell to cell, thus suggesting a “universal” shape of the size distribution. However, we still understand relatively little regarding how these simple principles emerge (Ho and Amir, 2015; Harris and Theriot, 2016; Wallden et al., 2016; Osella et al., 2017). For example, there is a debate on whether near-adder behavior could emerge from specific mechanisms or molecular circuits (Taheri-Araghi et al., 2015; Harris and Theriot, 2016), as a byproduct of chekpoint control of cell-cycle progression (Adiciptaningrum et al., 2015; Wallden et al., 2016), or as a consequence of external constraints or trade-offs (Osella et al., 2017).

Comprehensive and precise quantitative methods are necessary in both data analysis and theory to deal with the new data. Broadly, the open question is how much a mechanism can be isolated and specified with available data. The simplest theoretical framework for cell-cycle control describes the cell cycle as a discrete-time process, relating the measured variables (size, interdivision times, etc.) across generations (Amir, 2014; Campos et al., 2014; Taheri-Araghi et al., 2015; Grilli et al., 2017). We have recently shown the general existing equivalence between this simple discrete-time formalism to more detailed continuous-time models describing the division process by a division rate varying with cell state and time (Grilli et al., 2017). In the limit where deviations around the mean initial size (or interdivision time) are small (as first proposed in Amir, 2014), we have shown explicitly how the resulting “linear-response” framework describes a wide range of division control mechanisms and characterizes with remarkable precision the available experimental data. These results make the linear-response framework perfectly suitable to model cell-cycle control in *E. coli* given the available datasets.

However, studies are usually restricted to a subset of the measured variables. Most importantly, while single-cell growth rate clearly fluctuates (Wang et al., 2010), very few studies have addressed the consequences of these fluctuations on cell division control, and the studies that did started from very specific assumptions (Osella et al., 2014; Wallden et al., 2016; Logsdon et al., 2017). More specifically, previous studies adopting the linear-response framework typically neglected cell-to-cell variability in growth rates.

Here, we extend this linear-response framework to incorporate growth rate fluctuations, and we show how it can be used in general to evaluate exhaustively all the possible correlations and fluctuations in the data. We use this systematic approach to evaluate jointly all the homeostatic and noise-generating couplings measured in different experimental studies, and to connect correlation patterns with possible mechanisms underlying cell division.

## 2. Data sets

We tested our theoretical considerations and their implications on the analysis of empirical data with *E. coli* single-cell growth division data from Kiviet et al. (2014), Taheri-Araghi et al. (2015), Kennard et al. (2016), and Wallden et al. (2016). Scripts and formatted data are available with the authors. A detailed list of the growth conditions is available in the Supplementary Information of Cadart et al. (2017).

## 3. Linear-response framework for cell growth-division fluctuations

### 3.1. General features of fluctuations of cell size and growth rate

This section describes the main model assumptions and definitions. We assume exponential growth (Campos et al., 2014; Iyer-Biswas et al., 2014b; Osella et al., 2014; Taheri-Araghi et al., 2015) of cell size *V*(*t*) = *V*(0)exp(α*t*), where α is the single-cell growth rate. A cell divides at size *V*_*f*_ = *V*_0_exp(ατ_*d*_), where *V*_0_ is the size at birth and τ_*d*_ is the division time. We neglect the fluctuations around binary fission or the process of filamentation and recovery (Osella et al., 2014), thus cell size at division *V*_*f*_ is equally partitioned between the two daughter cells in our description. Given the assumption of exponential growth, it is useful to introduce the logarithmic size *q*(*t*) = log(*V*(*t*)/V^*^). With this notation, the exponential growth translates into a linear relationship *q*(*t*) = *q*(0)+α*t*. In the definition of *q*, we introduced a size scale V^*^, which in principle can be an arbitrary scale, to make the argument of the logarithm dimensionless. A particularly useful choice is to define $V^{*}=\langle V_{0}\rangle$, so that, at least in the small noise limit, 〈*q*(0)〉 = 0 independently of the condition (Grilli et al., 2017).

**Figure 1:**
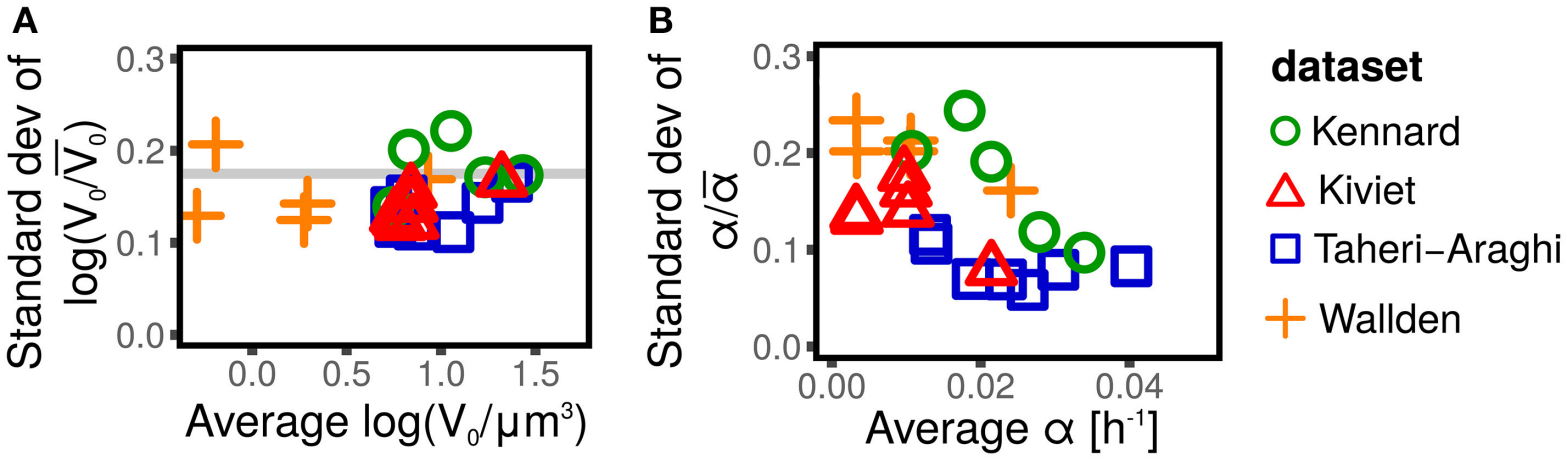
Scaling of size fluctuations, quantified by log-initial size log*V*_0_, and single-cell growth rate α for different datasets (symbols and colors) and conditions. Each symbol represents an average over all single cells available in the dataset (defined by shape and color) for a specific strain in a specific condition. **(A)** Shows that the standard deviation of the rescaled log-initial size log(*V*_0_/〈*V*_0_〉), is constant across conditions and experiments, and does not depend on the average log-initial size 〈log*V*_0_〉. This fact is a consequence of the collapse of initial size distribution (Taheri-Araghi et al., 2015; Kennard et al., 2016; Grilli et al., 2017), since the standard deviation log(*V*_0_/〈*V*_0_〉) is a function of the coefficient of variation of the initial size only (Grilli et al., 2017). **(B)** Shows the standard deviation of the rescaled growth rate α/〈α〉 (which corresponds to the coefficient of variation of α). This quantity is approximately constant for some datasets (e.g., Taheri-Araghi et al., 2015, blue squares), suggesting a collapse of the growth rate distribution (Taheri-Araghi et al., 2015), while it shows a decreasing trend in others (e.g., Kennard et al., 2016, green circles).

The distribution of cell size at birth gives a static picture of cell-size fluctuations. It is well accepted (Taheri-Araghi et al., 2015; Kennard et al., 2016; Grilli et al., 2017) that the distributions of size at birth (or, equivalently, at division) obtained for different strains and conditions collapse when rescaled by their average. This collapse is the consequence of the existence of a unique size-scale parameter of cell size control that varies across conditions (Kennard et al., 2016; Grilli et al., 2017). A consequence of the collapse is that the expectation of any function of the rescaled size at birth *V*_0_/〈*V*_0_〉 is constant across conditions. Figure 1 shows that the variance of log*V*_0_/〈*V*_0_〉 (which is by definition equal to $\sigma_{q}^{2}$) is constant across conditions, as predicted by the collapse of the size distribution.

Growth rates fluctuations are usually neglected. Contrarily to the well accepted collapse of the size distributions (Giometto et al., 2013; Taheri-Araghi et al., 2015; Kennard et al., 2016; Grilli et al., 2017), there is no consensus on whether the distribution of the single-cell growth rates collapse when rescaled by the average growth rate, with some studies reporting the collapse (Taheri-Araghi et al., 2015) and others showing the opposite (Kennard et al., 2016). Figure 1 shows that the coefficient of variation of the growth rate appears to be constant, independently of the condition and the average growth rate (consistently with the collapse hypothesis) for some datasets (Taheri-Araghi et al., 2015), while it shows a decreasing trend for others (Kiviet et al., 2014; Kennard et al., 2016; Wallden et al., 2016). A possible explanation of these differences could lay in the growth mode of the different experiments (microcolonies in agar or polyacrylamide, wide microfluidics channels, single-file microfluidics channels, etc). Theoretical efforts to provide a rationale of growth rate distributions are available (Iyer-Biswas et al., 2014b; Pugatch, 2015; De Martino et al., 2016; De Martino et al., 2017).

Independently of the controversy about the collapse of the growth rate distributions, it is clear from Figure 1, that the fluctuations of the growth rates are not negligible. The coefficients of variation are in the range 0.1–0.3, which is comparable with the typical fluctuations of the sizes at birth, whose CVs are of the order of 0.2 (Amir, 2014; Grilli et al., 2017).

### 3.2. Linear-response framework in presence of growth rate fluctuations

Since the coefficient of variation of both sizes and growth rates are around 0.2, it is reasonable to assume that the size-control mechanism can be expanded around the average size and average growth rate (Amir, 2014; Grilli et al., 2017). This maps the problem into a discrete-time linear response framework. This section generalizes the usual linear response approach to include single-cell growth rates fluctuations, with the goal of disentangling how size and growth rate fluctuations are connected.

We use a discrete-time description, focusing on a single-cell at a given generation *i*. This cell has initial size $V_{0}^{\left(i\right)}$, which corresponds to a log-size at birth $q_{0}^{\left(i\right)}$, a growth rate α^(*i*)^ and divides at a log-size $q_{f}^{\left(i\right)}$ after a time $\tau_{d}^{\left(i\right)}$. These variables are connected by the relation

(1) $$\begin{array}{r} q_{f}^{\left(i\right)}=q_{0}^{\left(i\right)}+\alpha^{\left(i\right)}\tau_{d}^{\left(i\right)}\;. \end{array}$$

We also indicate the net logarithmic multiplicative growth (sometimes referred to as elongation) by

$$G^{\left(i\right)}=\alpha^{\left(i\right)}\tau_{d}^{\left(i\right)}=\log\left(V_{f}^{\left(i\right)}/V_{b}^{\left(i\right)}\right)=q_{f}^{\left(i\right)}-q_{0}^{\left(i\right)}\;.$$

Here, α^(*i*)^ is a random variable with mean 〈α〉 (which depends on the condition) and variance $\sigma_{\alpha }^{2}$. We assume that individual cells maintain the same growth rate for a whole cell cycle (this approximation corresponds to how growth rate distributions are often evaluated in empirical data). The growth rates of subsequent generations are drawn from a common (Gaussian) distribution, but may be correlated across generations, with Pearson correlation coefficient ρ. An alternative approach to model growth fluctuations would consist in modeling the stochastic fluctuations of α^(*i*)^ in continuous time (Iyer-Biswas et al., 2014a,b). The more minimalistic discrete-time approach that we employ can be seen as a coarse-graining of an appropriate continuous-time model. We also note that the discrete-time modeling approach is equivalent to a continuous-time description for single-exponential growth (Grilli et al., 2017; Ho et al., 2018), but in presence of a growth rate fluctuating in continuous time, this equivalence does not hold anymore. For the small fluctuations observed in the data, the two approaches are qualitatively equivalent and likely quantitatively not distinguishable given the available statistical power.

We introduce the variable fluctuations

(2) $$\begin{array}{r} \begin{array}{cc} \delta G^{\left(i\right)} & :=G^{\left(i\right)}-\langle G\rangle \\ \delta \tau_{d}^{\left(i\right)} & :=\tau_{d}^{\left(i\right)}-\langle \tau \rangle \\ \delta \alpha^{\left(i\right)} & :=\alpha^{\left(i\right)}-\langle \alpha \rangle \\ \delta q_{0}^{\left(i\right)} & :=q_{0}^{\left(i\right)}-\langle q_{0}\rangle \;. \end{array} \end{array}$$

Under the above assumptions, the evolution equations for the fluctuations of the three main variables can be written as

(3) $$\begin{array}{r} \begin{array}{cc} \frac{\delta G^{\left(i\right)}}{\sigma_{G}} & =-\lambda_{Gq}\frac{\delta q_{0}^{\left(i\right)}}{\sigma_{q}}-\lambda_{G\alpha }\frac{\delta \alpha^{\left(i\right)}}{\sigma_{\alpha }}+\nu_{G}^{\left(i\right)}\\ \frac{\delta \tau_{d}^{\left(i\right)}}{\sigma_{\tau }} & =-\lambda_{\tau q}\frac{\delta q_{0}^{\left(i\right)}}{\sigma_{q}}-\lambda_{\tau \alpha }\frac{\delta \alpha^{\left(i\right)}}{\sigma_{\alpha }}+\nu_{\tau }^{\left(i\right)}\;, \end{array} \end{array}$$

where λ_*XY*_ are linear coupling parameters (analogous to “susceptibilities”) between measured variables. Such linear couplings are central in comparing this theory to experimental data. $\nu_{\tau }^{\left(i\right)}$ and $\nu_{G}^{\left(i\right)}$ are zero-mean random variables representing cell-to-cell variability (“noise”). Previous approaches neglecting cell size fluctuations assume δα = 0. In order to be solved, Equation 3 requires the specification of how the fluctuations δα^(*i*)^ are related to the fluctuations of logarithmic size $\delta q_{0}^{\left(i\right)}$. We assume that generally the individual growth rate may depend on the initial size, i.e., within a linear-response framework,

(4) $$\begin{array}{r} \frac{\delta \alpha^{\left(i\right)}}{\sigma_{\alpha }}=-\lambda_{\alpha q}\frac{\delta q_{0}^{\left(i\right)}}{\sigma_{q}}+\nu_{\alpha }^{\left(i\right)}\;. \end{array}$$

The values of these coupling constants can be associated to specific mechanisms of size control (Amir, 2014; Grilli et al., 2017). For instance a timer corresponds to λ_τ*q*_ = 0 and λ_τα_ = 0, while a sizer mechanism implies λ_*Gq*_σ_*G*_/σ_*q*_ = 1 and λ_*Gα*_ = 0. A standard adder corresponds instead to λ_*Gq*_σ_*G*_/σ_*q*_ = 1/2 (Amir, 2014; Grilli et al., 2017).

This general framework can be used to evaluate the direct couplings between fluctuations of birth size, growth rate and division time. In particular, all the couplings λs can be evaluated from the Pearson correlations of these variables or from the slope of conditional averages. The two methods are equivalent if the slopes are estimated using least-squares method. Indeed, assuming a relationship *y* = β^*^x+noise, and performing a linear regression, the least-square estimate of beta is β = cov(*x, y*)/var(*x*). Any discrepancy of the values of the coupling parameters obtained in these two different ways should be considered as a signature of the violation of the linear-response regime (Cadart et al., 2017). For instance, by multiplying both sides of Equation (4) by δ*q*_0_/σ_*q*_ and averaging over the fluctuations, it is easy to obtain (see Supplementary Information)

(5) $$\begin{array}{r} c_{\alpha q}=-\lambda_{\alpha q}\;, \end{array}$$

where *c*_α*q*_ is the Pearson correlation between the growth rate α and the log-size *q*. Similarly, multiplying by δ*q*/σ_*q*_ Equation (3) and averaging gives

(6) $$\begin{array}{r} c_{\tau q}=-\lambda_{\tau q}-\lambda_{\tau \alpha }c_{\alpha q}=-\lambda_{\tau q}+\lambda_{\tau \alpha }\lambda_{\alpha q}\;. \end{array}$$

Finally, multiplying the same equation by δα/σ_α_ and averaging over fluctuations gives

(7) $$\begin{array}{r} c_{\tau \alpha }=-\lambda_{\tau q}c_{\alpha q}-\lambda_{\tau \alpha }=\;-\lambda_{\tau \alpha }+\lambda_{\tau q}\lambda_{\alpha q}\;. \end{array}$$

A detailed comparison between the two ways to evaluate the couplings in empirical data is shown in the Supplementary Information of Cadart et al. (2017). The very small discrepancies found reinforce the ideas that the linear-response framework is effective.

The last two equations clearly show that the presence of a non-zero coupling between single-cell growth rate and birth size induces a non-trivial dependence of the correlations from all the coupling parameters. In other words, when the coupling between division time and cell size is estimated uniquely from *c*_τ*q*_ (Amir, 2014; Grilli et al., 2017), one is measuring a combination of two different effects: the direct coupling between division time and size at birth and an indirect coupling mediated by growth rate fluctuations.

Direct and indirect effects can be disentangled by solving Equations (6) and (7) to calculate λ_τ*q*_ and λ_τα_ in terms of measurable covariances,

(8) $$\begin{array}{r} \lambda_{\tau q}=-\frac{c_{\tau q}-c_{\alpha q}c_{\tau \alpha }}{1-c_{\alpha q}^{2}}\;, \end{array}$$

and

(9) $$\begin{array}{r} \lambda_{\tau \alpha }=-\frac{c_{\tau \alpha }-c_{\alpha q}c_{\tau q}}{1-c_{\alpha q}^{2}}\;. \end{array}$$

**Figure 2:**
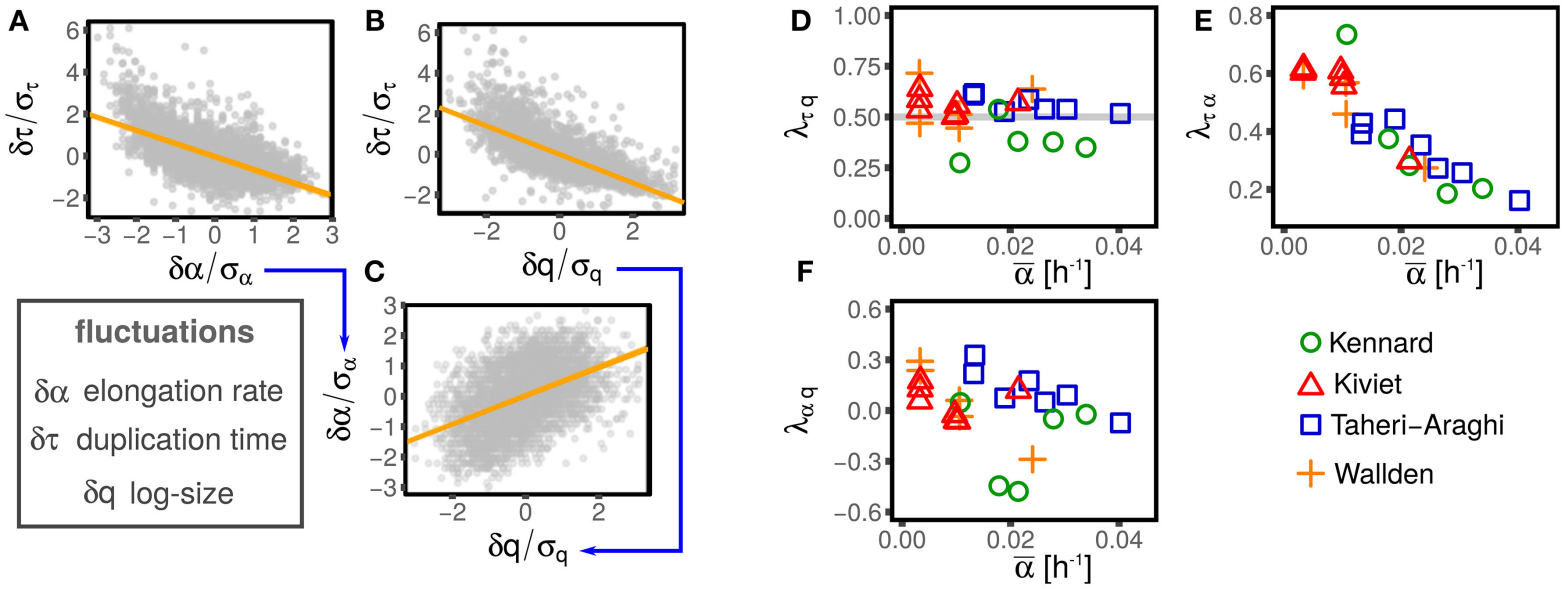
Illustration of the linear-response framework of size control, including growth rate fluctuations. **(A,B)** Show the dependency of the interdivision time fluctuations from fluctuations of logarithmic initial size and growth rate for one dataset (Wallden et al., 2016 intermediate growth rate). Since growth rate and size fluctuations are not independent **(C)**, the slopes observed in **(A,B)** are determined by a combination of the coupling between size fluctuation and interdivision time (with strength λ_τ*q*_, see Equation 2) and the effect of growth rate fluctuation on interdivision time (with strength λ_τα_, see Equation 2). The method described in the text disentangles direct and indirect effect on the slopes of **(A–C)** to obtain the direct couplings between τ, α, and *q*, which are shown in **(D–F)**, for different datasets (symbols and colors) and conditions. Interestingly, all the possible couplings are non-zero. Current models do not describe these nontrivial correlations, which poses a challenge to future models.

Figure 2 illustrates this procedure over one example dataset.

The main assumption of the above modeling framework is that all the relations between the variables are linear. Deviations from linear response are not captured, but in the data these are fairly small (see Figure 2). Additionally, it is difficult to evaluate precisely the non-linear behavior from data because the statistics decreases radically for large fluctuations where such non-linear trends are stronger. Given the statistical power available from existing datasets, it is typically not possible to discriminate non-linear terms from stochastic fluctuations (Grilli et al., 2017).

It has previously been noted that measured Pearson correlations between observed variables in such experiments are very sensitive to (experimental and biological) noise (Eun et al., 2018), so that it is not simple to reconstruct mechanisms from correlations. Our approach, which directly tackles this issue, has two main advantages. First, it subtracts the contributions of indirect correlations and only measures direct couplings. Second, the coupling constants are insensitive to noise, because they are defined as linear slopes of conditional averages, and therefore robust to experimental noise. This is supported by the analysis of Cadart et al. (2017) mentioned above, verifying the equivalence between theory coupling constants measured directly and their expressions in terms of covariances. Indeed, this equivalence not only supports the validity of the linear regime, but also reinforces the idea that scatter in the data (due e.g., to experimental noise) does not bias too much the coupling constants, when they are estimated from the covariances.

We also note that the choice of parameters in Equations (3) and (4) is not unique. There are several alternative (equivalent) choices of fluctuating variables, linked by the condition that *G* = ατ: α, τ, *q*; α, *G, q*; *G*, τ, *q*. Each triplet carries a set of linear-response coupling constants between pairs of variables that can be mapped to the ones defined above.

## 4. Results

### 4.1. All the possible couplings between measured variables are non-zero in empirical datasets

One might expect that not all the possible couplings are different from zero, supporting the simplifying assumptions made by most studies. Figure 2 shows instead that all the independent couplings λ_τ*q*_, λ_τα_, and λ_α*q*_ are in fact not negligible.

**Figure 3:**
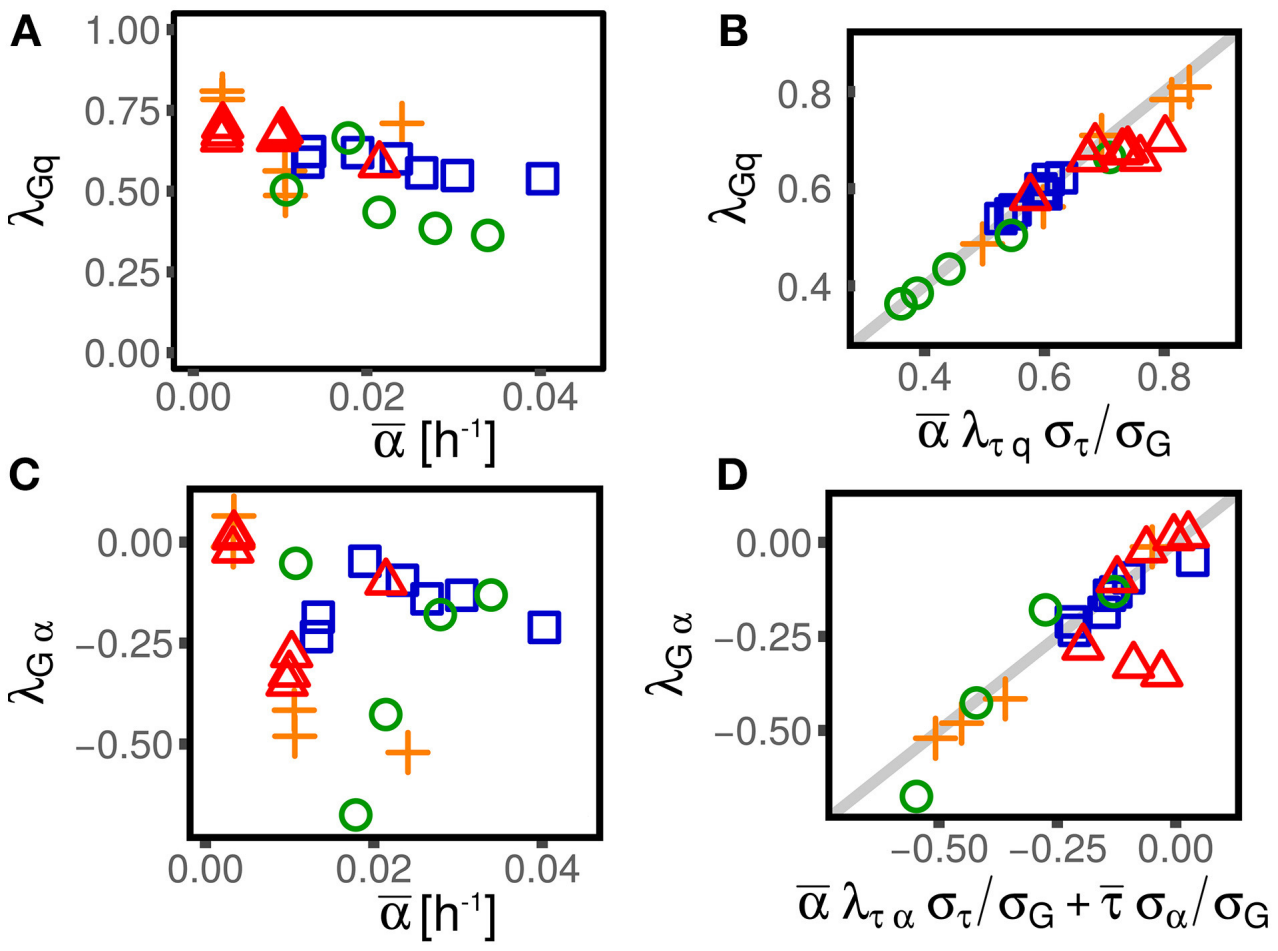
Alternative parameterizations of cell-size control linear response are consistent across different experimental datasets and conditions (symbols and colors are the same as in Figure 1). One can parametrize the effect of size and growth rate fluctuations on interdivision time, or, alternatively, their effect on the final size (or the elongation *G*). **(A,C)** Show the effect of log-size fluctuation on elongation (with strength λ_*Gq*_, see Equation 3) and the effect of growth rate fluctuation on interdivision time (with strength λ_*Gα*_). The linear framework predicts consistency relations between the alternative parameterizations **(B,D)**, which are verified across datasets and conditions in the plots in the right panel.

The existence of nonzero couplings between division time and growth rate λ_τα_ or, equivalently, elongation and growth rate λ_*Gα*_ (see next section and Figure 3) suggests that cell size control depends in a non-trivial way on growth rate fluctuations. Moreover, the existence of a coupling between growth rates and cell size (measured by the coefficient λ_α*q*_), affects the observed correlation between division time and size at birth *c*_τ*q*_ (see Equation 6), or, equivalently, the slope of the size-growth plot *c*_*Gq*_ (Skotheim, 2013), which is normally used to evaluate the strength of the size control. Different values of these correlations are associated with different strengths of cell size control (Skotheim, 2013; Osella et al., 2014), and, more indirectly, to different cell size control mechanisms (Amir, 2014; Grilli et al., 2017). The presence of non-zero couplings between interdivision time and growth rate and between growth rate and size effectively reduces or increases the observed strength of homeostasis, and might be a signature of the mechanisms effecting such control.

### 4.2. The linear-response framework allows to define consistency relations

As discussed above, the framework provides equivalent alternative descriptions for the interdivision time τ or the net growth *G* as state variables. These two parameter settings are equivalent under the linear-control assumption. This section deals with the mapping between these parametrizations, and reports consistency tests defined by the expected relations using empirical data.

The consistency relations can be obtained from the relation *G*^(*i*)^ = α^(*i*)^τ^(*i*)^, which, under the linearization assumption reduces to (see Supplementary Information)

(10) $$\begin{array}{r} \delta G^{\left(i\right)}=\langle \tau \rangle \delta \alpha^{\left(i\right)}+\langle \alpha \rangle \delta \tau^{\left(i\right)}\;. \end{array}$$

Using this relation together with Equation (3), one obtains (see Supplementary Information)

(11) $$\begin{array}{r} \lambda_{Gq}=\langle \alpha \rangle \lambda_{\tau q}\frac{\sigma_{\tau }}{\sigma_{G}} \end{array}$$

and

(12) $$\begin{array}{r} \lambda_{G\alpha }=\langle \alpha \rangle \lambda_{\tau \alpha }\frac{\sigma_{\tau }}{\sigma_{G}}+\langle \tau \rangle \frac{\sigma_{\alpha }}{\sigma_{G}}\;. \end{array}$$

An important consequence of Equation (12) is that a nonzero λ_*Gα*_ (or λ_τα_) could appear even if the corresponding coupling λ_τα_ (or λ_*Gα*_) was null. For instance, if the coupling λ_τα_ is negligible, indicating that the interdivision time is independent from the single-cell growth rate, one would still observe a nonzero λ_*Gα*_. In fact, in that case, the division time would be independent of the growth rate and therefore, in the same amount of time, cells growing faster would grow more than slow-growing cells. Interestingly, this is not the case. Both λ_τα_ (see Figure 2) and λ_*Gα*_ are in fact different from zero (see Figure 2), suggesting that some specific mechanism must be at play in determining their value.

Figure 3 shows that these consistency relations are verified in the data. This result shows that the generalization of the linear-response framework to include growth rate fluctuations can correctly describe the data. As previously discussed, small deviations from these relations could be due to the presence of non-linearities that cannot be captured by the theoretical framework. However, non-linearities can be appreciated only in presence of large fluctuations (Grilli et al., 2017). Also note that while these deviations could be the signature of not-yet-understood biological mechanisms, they could also come from measurement, segmentation or tracking errors associated to specific experimental and image-analysis protocols.

### 4.3. Growth rate fluctuations can confound standard tests of division control mechanisms, the case of “grower” vs. “timer”

**Figure 4:**
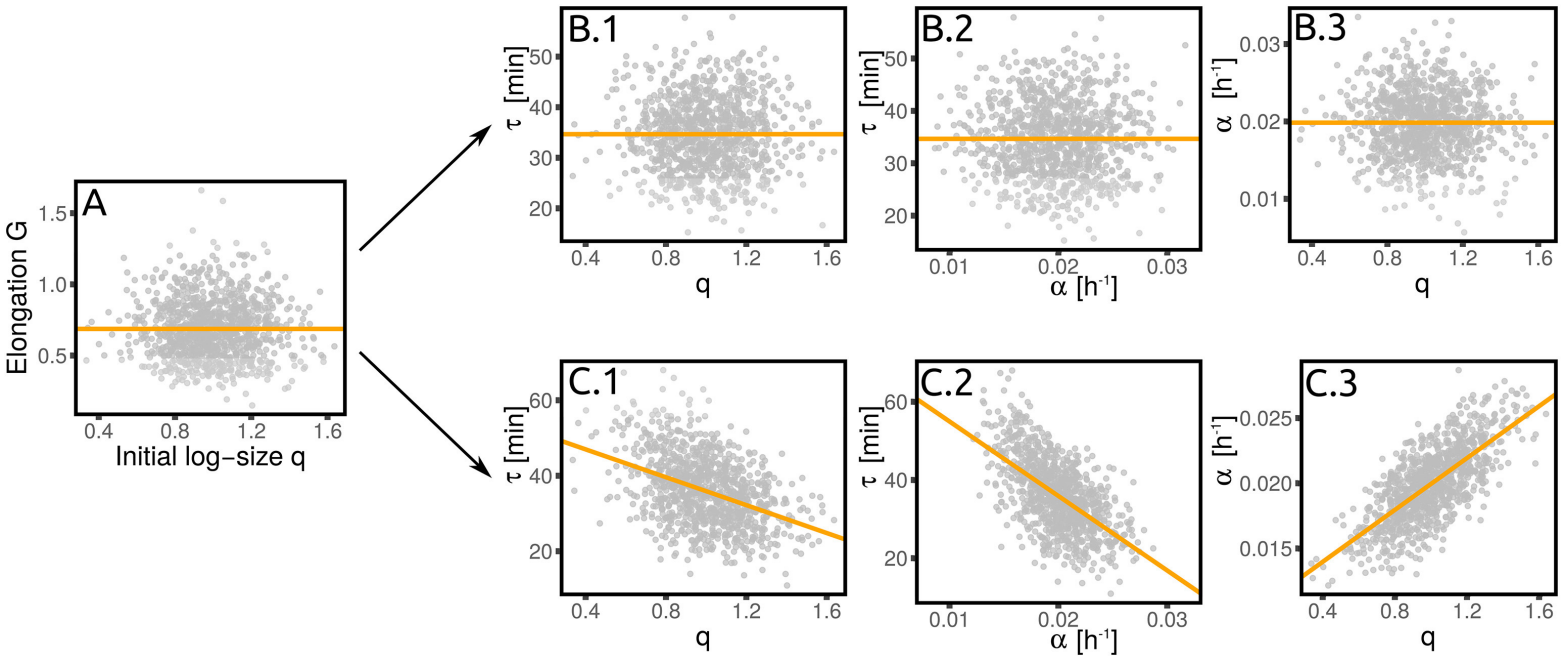
A grower is not a timer. The absence of a correlation between elongation and initial size **(A)** does not necessarily imply a timer **(B)**, where the interdivision time is independent of the initial size. **(C)** Shows the alternative scenario (the grower), where the growth rate depends on the initial size. This correlation, together with the independence of the elongation from the growth rate, implies a dependence of the interdivision time on both initial size and growth rate. Both panels are obtained from numerical simulations of the corresponding linearized model with σ_α_/〈α〉 = 0.2 and $\sigma_{q}^{2}=0.2$.

A standard simple analysis to understand what kind of cell division control is in place is based on size-growth plots (Skotheim, 2013) and more generally on measures of correlation between cell elongation and initial size. Since growth rate fluctuations are typically neglected, a value of λ_*Gq*_ = 0, i.e., no correlation between elongation and size at birth (Figure 4A), is usually interpreted as the signature of a “timer.” However, in presence of growth rate fluctuations the value of this coupling parameter alone is not enough to pinpoint a single mechanism. In fact, λ_*Gq*_ = 0 can be the actual result of a timer, i.e., λ_τ*q*_ = 0 which then requires that λ_τα_ = 0 (Figure 4B). However, the relevant variable that is actually uncoupled to size could be the net growth (elongation) itself, rather than the doubling time and the growth rate. In this case, one has a “grower” (Figure 4C), for which again λ_*Gq*_ = 0 and λ_*Gα*_ = 0. For a grower, λ_τ*q*_ = 0, but λ_τα_ = −〈τ〉σ_α_/(〈α〉σ_τ_). Importantly, if λ_α*q*_≠0, then the correlation between τ and *q* (*c*_τ*q*_) is also different from zero in a grower. This illustrative example emphasizes the importance of keeping into account growth rate fluctuations in order to correctly interpret empirical correlations patterns. It also shows how the linear-response framework proposed here can be used to delineate the correct interpretation. A more general decomposition of contribution of growth vs. timing coupling to size control is discussed in Cadart et al. (2017).

We now proceed to describe two situations where our analysis points directly to specific mechanisms contributing to the complexity of the observed correlation patterns.

### 4.4. Mother-daughter growth rate correlations explain the prevalent negative coupling between growth rates and cell size observed in data

Since the framework describes exhaustively the measured correlations in the datasets, it can be used to explore signatures of mechanisms by which division is coupled to size. This section provides a rationale for the emergence of a non-zero coupling between single-cell growth rate and size at birth λ_α*q*_ observed in most datasets. In particular, we show that a negative λ_α*q*_ may emerge from the presence of a correlation between the growth rates across generations.

**Figure 5:**
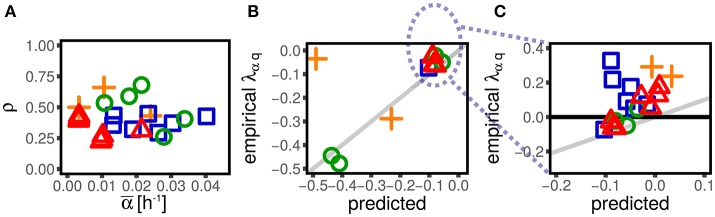
Mother-daughter correlations in growth rate induce negative values of λ_α*q*_. **(A)** Shows the correlation between mother and daughter growth rate across datasets (symbols and colors, see Figure 1) and conditions. All the datasets display a significant positive correlation. Panel shows the value of λ_α*q*_ predicted from our theory assuming the empirical mother-daughter growth rate correlations (see Equation 14), compared to the empirical ones for negative values of λ_α*q*_. The assumed mechanism correctly predicts negative values of λ_α*q*_, but the prediction cannot capture the datasets with positive values of λ_α*q*_, which are shown in **(C)** (not visible in **B**).

Figure 5 shows the Pearson correlation ρ between the mother and the daughter(s) growth rates in several data sets. We computed the correlation using both the daughters (when available in the dataset). Some previous studies have reported, or assumed, small mother-daughter correlations in growth rate (Wang et al., 2010; Eun et al., 2018), while others have found larger ones (Wallden et al., 2016). An important result of the present analysis is that this correlation is never negligible in the analyzed data sets and appears very conserved across conditions and experimental setup. Specifically, all the datasets considered here show a significantly positive correlation, which does not seem to depend strongly on the condition and takes values around 0.3. Variations of single-cell growth rates can be both due to an external origin (e.g., local variation of nutrients in an agar experiment) or inherent to the mechanisms of growth (e.g., from fluctuations in gene expression Iyer-Biswas et al., 2014a). In both cases, one may expect the presence of positive correlation across generations.

We can model mother-daughter correlations in growth rate as a linear constraint on the single-cell growth rates over generations

(13) $$\begin{array}{r} \delta \alpha^{\left(i+1\right)}=\rho \delta \alpha^{\left(i\right)}+\sigma_{\alpha }\nu_{\alpha }^{\left(i\right)}\;. \end{array}$$

This equation substitutes Equation (4), and must be considered together with Equation 3. The question we address is whether inter-generational correlations give rise to a non-zero coupling λ_α*q*_ between single-cell growth rate and size.

Since we are considering a fully linear framework, a non-zero coupling λ_α*q*_ can be directly mapped into a non-zero correlation between single-cell growth rate and size *c*_α*q*_ via Equation (5). We find that a non-zero correlation *c*_α*q*_ emerges in presence of a non-zero correlation of growth rate across generation and a non-zero coupling λ_*Gα*_. In the Supplementary Information we obtain the relation

(14) $$\begin{array}{r} \lambda_{\alpha q}=-c_{\alpha q}=\rho \frac{\lambda_{G\alpha }}{1-\rho \left(1-\lambda_{Gq}\sqrt{\frac{\sigma_{G}^{2}}{\sigma_{q}^{2}}}\right)}\sqrt{\frac{\sigma_{G}^{2}}{\sigma_{q}^{2}}}\;. \end{array}$$

From the above equation we observe that, for positive ρ and negative λ_*Gα*_ (the case of empirical data) only negative couplings λ_α*q*_ can emerge. Hence, mother-daughter growth rate correlations provide a rationale for this widespread negative coupling.

Figure 5 shows that the prediction of Equation (14) quantitatively reproduces the values of λ_α*q*_ for the datasets that display negative values of this coupling. On the other hand, some datasets have weakly positive couplings λ_α*q*_, which cannot be reproduced via this mechanism. This suggests that some other unknown mechanisms might play a role in coupling growth rates and sizes.

### 4.5. An adder model captures the experimental correlation patterns only if added size depends on single-cell growth rate

This section explores how the observed negative values for the coupling λ_*Gα*_ emerge from a simple extension of the adder model that explicitly includes growth rate fluctuations. According to the adder model (Amir, 2014; Taheri-Araghi et al., 2015), the size at division *V*_*f*_ is given by

(15) $$\begin{array}{r} V_{f}=\left(V_{0}+\Delta \right)e^{\nu }\;, \end{array}$$

where Δ is the added size and ν is a noise term. We also know that the average size 〈*V*_0_〉 has an exponential dependence on the average growth rate 〈α〉, which is often referred to as Schaechter's law (Schaechter et al., 1958; Taheri-Araghi et al., 2015; Kennard et al., 2016; Si et al., 2017). Since for steadily dividing cells the average size at birth is equal to the added size, the exponential dependence of the average size on the average growth rate also implies that the average added size must have an exponential dependence on the average growth rate.

Equation (15) does not take into account fluctuations of the growth rate, and there are several possibilities to extend it. Schaechter's law can be seen as a constraint, as the added size averaged over fluctuations of the growth rate has to scale nearly exponentially with the average growth rate. Two possible extreme scenarios can be disentangled with the data. The first scenario assumes that in a given growth condition the added size does not depend on the fluctuation of the growth rate, i.e.,

(16) $$\begin{array}{r} \Delta =S_{0}\exp\left(\langle \alpha \rangle T\right)\;. \end{array}$$

In such case, two cells with different individual growth rates, but growing in the same condition, will add (on average) the same size. In the opposite extreme scenario, the added size depends on the single-cell growth rate, following Schaecter's law *even for small fluctuations*

(17) $$\begin{array}{r} \Delta =S_{0}\exp\left(\alpha T\right)\;. \end{array}$$

In this second scenario, cells in the same growth conditions growing at different growth rates will add on average different sizes. Conversely, cells growing under different conditions but having the same growth rate due to single-cell fluctuations will add (on average) the same size. Since fluctuations of α are small, in both cases the average added size 〈Δ〉 will scale exponentially with the average growth rate.

**Figure 6:**
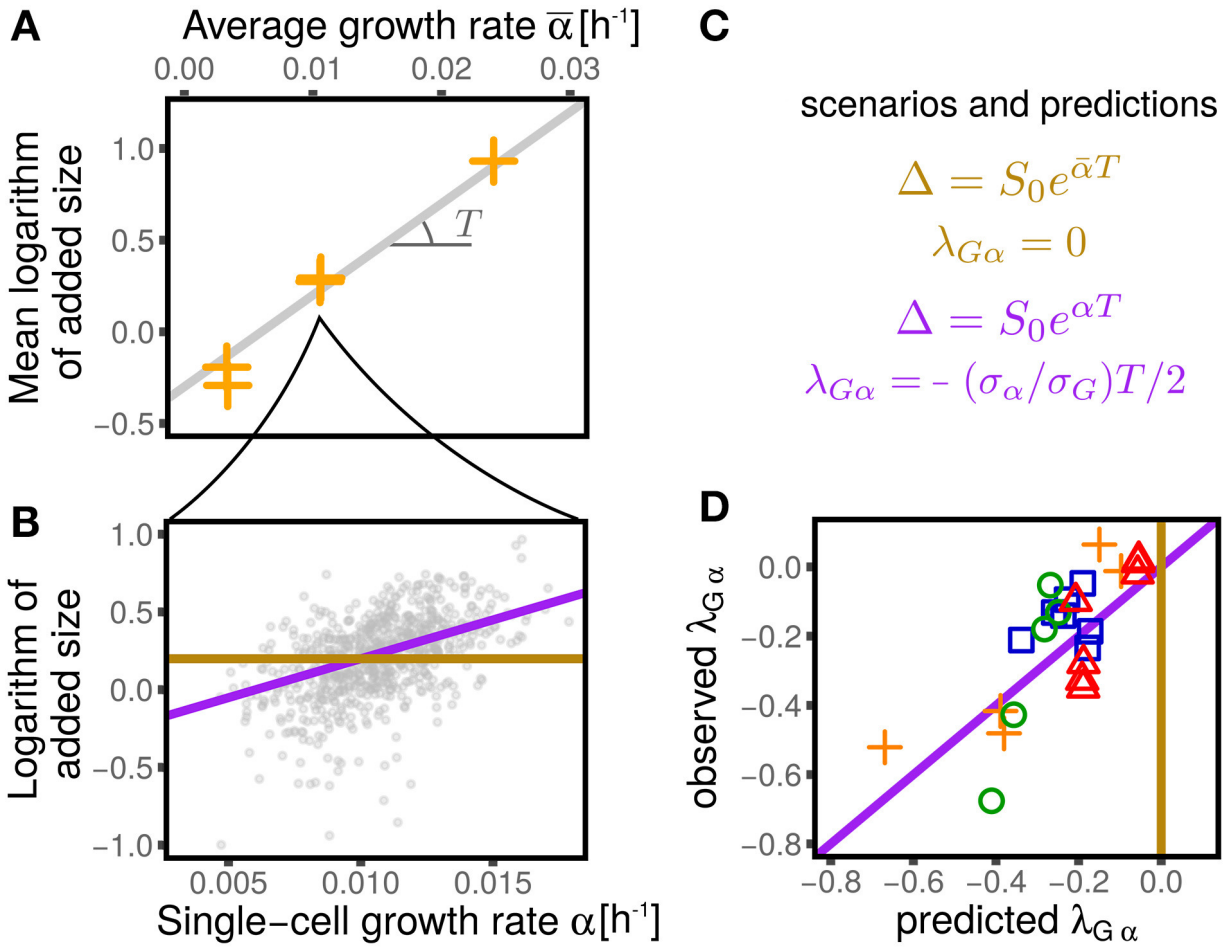
Dependency of added size on single-cell growth rates and effect on the values of λ_*Gα*_. **(A)** Shows that the average logarithmic added size is linear when plotted against the average growth rate (for all the conditions from Wallden et al., 2016). We consider two extreme scenarios for the logaritmic added size of individual cells: it could depend only on the average growth rate (gold line) or it could linearly grow with the single-cell growth rate (purple line), with the same slope observed in **(A)**. **(B)** Shows the results of the two scenarios for one condition (intermediate growth rate data set from Wallden et al., 2016). These two scenarios translate into two different predictions for the value of λ_*Gα*_, which are reported in panel **(C)** and tested in panel **(D)**. The non-zero values of λ_*Gα*_ are therefore a consequences of the dependence of the added size on the the single-cell growth rate.

This question was approached in Kennard et al. (2016), but the linear-response theoretical framework gives us the tools to reformulate it more precisely. Figure 6 shows how we proceed to test the two scenarios. For each dataset we estimated the parameter *T* (which can be related to the duration of the replication/segregation (“C+D”) period Donachie, 1968; Zheng et al., 2016; Si et al., 2017) from 〈logΔ〉 averaged across cells within each condition. Note that for both Equations (16) and (17), the slope of 〈logΔ〉 *vs* 〈α〉 is *T*. For each condition, one can then compare the relation between the (logarithmic) added size and the individual growth rate. Under the first scenario, the relation should show no dependency (a flat slope). Under the second scenario, one should instead expect a linear relation with slope *T* (where *T* is the mean timing from replication initiation to division, assumed to be nearly constant across conditions).

Under the assumption that fluctuations are small, one can linearize the models and compare the coupling coefficients. In the first case, one obtains λ_*Gα*_ = 0, while in the second case a non-trivial coupling arises with value

(18) $$\begin{array}{r} \lambda_{G\alpha }=\frac{\sigma_{\alpha }}{\sigma_{G}}\;-\;\frac{T}{2}. \end{array}$$

Figure 6 compares this prediction with the empirical values of this coupling parameter measured across datasets, obtaining a remarkable agreement. It is important to stress that the parameter *T*, which in turn determines the value of λ_*Gα*_, has been measured only using the dependence of average added size on the average growth rate across different conditions, without considering single-cell data. This procedure assumes that the parameters *S*_0_ and *T* are constant within each dataset across different conditions (Donachie, 1968; Si et al., 2017). Variation around Schaecter's law, which could arise for many reasons (Zheng et al., 2016; Si et al., 2017), can affect the values of these parameters. Precise estimates, or measurements of *T* can improve the prediction of Equation (18). Moreover, the variability around the adder mechanism (visible in the values of λ_*Gq*_ plotted in Figure 3) also affects the predictions.

## 5. Discussion and conclusions

With a long list of recent published studies, the current literature on cell division control remains fragmented in terms of conceptual tools and conclusions on the data (Osella et al., 2017).

This work proposes an extension of the linear-response framework to include growth rate fluctuations, and at the same time provides a comparative meta-analysis of different datasets. As such, we hope it could be useful to the community. The results lead us to propose the linear-response model as a basic common data-analysis framework for the recent (and future) growth division data obtained on bacteria.

Analyzing the experimental data, we found two important results. First, the linear response framework including growth rate fluctuations provides a remarkably good description of the data. Couplings are in all cases only moderately nonlinear (Grilli et al., 2017), and all the predicted self-consistency relations are verified to a satisfactory degree. This carries the important lesson that models where nonlinear couplings are involved might be true, but they would be hard to test with the currently available data. Second, we show that the linear correlation patterns store a wealth of information that is not yet fully appreciated. In particular, single-cell growth rates seem to have a complex effect on cell cycle progression and cell-division control.

The study of the linear coupling parameters between growth rate and size reveals an unexpected variability across experiments. This variability depends on the growth conditions and on the measured mother-daughter correlations in growth rate. Thus, the effect of growth rate fluctuations on cell size varies from one experiment to the other and appears, in some cases, to contribute to size correction. In contrast, the near-adder correlation for the net growth *G* and the dependency of interdivision time on initial size are robustly observed in all datasets, although quantitatively, they also vary across conditions.

Since all these linear-response effects are clearly testable, we believe that future efforts should be funneled into explaining these patterns comprehensively. Each particular model for a size control mechanism carries a set of constraints for the linear-coupling parameters, which are easily testable in the data. Such testing involves all the measured variables and correlations. Hence, our framework provides a comprehensive and stringent tool to test proposed mechanisms.

The question of specifying the mechanisms for cell division and cell-cycle progression remains largely open, and therefore could benefit from such a systematic tool. One main limitation of current studies is that they discard relevant information and only consider a subset of correlation patterns. Conversely, linear-coupling parameters allow to control all the measured correlations at once. In this work, we provided a proof of principle that this plan of action can be effective by studying two simple examples: (i) the role of mother-daughter correlations in size control, and (ii) the role of growth rate fluctuation in determining the added size.

In particular, we find that growth rate fluctuations are not consistent with an adder model where the added size does not depend on the single-cell growth rate. This could be an important clue on the mechanism underlying the adder behavior and could help selecting one of the currently proposed models (Ho and Amir, 2015; Taheri-Araghi et al., 2015; Harris and Theriot, 2016; Wallden et al., 2016).

Finally, we address the coherence between different experimental data sets. The discrepancies we found could be due to biological factors, but also to possible sources of experimental noise and systematic errors in the data. Current experimental and data-analysis pipelines are different in both the segmentation and tracking steps. This problem is largely disregarded and in particular we know very little about possible induced biases in the measured correlations, which would lead to different conclusions on the mechanistic aspects. As end-users of the data, we propose our pipeline, i.e., comparing all measurable linear correlations as a useful testing ground for different datasets. In our analysis, we have noticed different trends depending on the experimental laboratory, and growth device, as well as outlier points where possibly the growth conditions were not steady or the analysis had problems. Interestingly, while some observations are robust, others are more erratic across datasets. Beyond this, we can just encourage the experimentalists to adopt common protocols and shared comparison pipelines in future studies. It would be sufficient to define a well defined “reference experiment” with fixed strain, growth device, and growth conditions that each data set needs to share, in order to make precise comparisons possible.

## Author contributions

MCL and JG planned the study. JG, MCL, and MO developed the modeling framework, JG, CC, and GM, analyzed data. JG and MCL outlined and wrote the paper. MO, CC, and GM contributed with scientific discussion and critical reading.

### Conflict of interest statement

The authors declare that the research was conducted in the absence of any commercial or financial relationships that could be construed as a potential conflict of interest.
