# Supplementary material for: The Empirical Fluctuation Pattern of E. coli Division Control
Source: Front Microbiol. 2018 Jul 30;9:1541. doi: 10.3389/fmicb.2018.01541 (PMC6077223; doi:10.3389/fmicb.2018.01541)
Supplement: Supplementary file 1 [file Presentation_1.pdf]

Supplementary Information for Grilli *et al.* "The empirical fluctuation pattern of *E. coli* division control"

We assume that the final volume (in a cell cycle or subperiod) is a result of a size-dependent growth and timing

$$V_f = V_0 \exp\left(\alpha(V_0)\tau(V_0,\alpha)\right) \ , \tag{A1}$$

where V_x is volume and α and τ quantify the duration and growth in the period (or cell cycle) and they are both random variables. Note that, formally, there is no hypothesis of exponential growth rate, just that growth during a period is represented as en effective exponential growth (this is formally always possible, and physically it is justified by the mean time traces).

$$q_f - q_0 = G(V_0) + \nu \tag{A2}$$

where ν is a noise term, $q_x = \log(V_x)$ and $G(V_0) = \alpha(V_0)\tau(V_0)$ and $q_f - q_0 = \log \frac{V_f}{V_0}$ so the previous equation is just the size-growth plot.

A. Notation and general response theory

Introducing the notation

$$\delta q = q_0 - \langle q_0 \rangle \tag{A3}$$

and

$$\delta\alpha = \alpha - \langle \alpha \rangle \ , \tag{A4}$$

we can write

$$\frac{\delta\tau_d^{(i)}}{\sigma_\tau} = -\lambda_{\tau q}\frac{\delta q_0^{(i)}}{\sigma_q} - \lambda_{\tau\alpha}\frac{\delta\alpha^{(i)}}{\sigma_\alpha} + \nu_\tau^{(i)} \tag{A5}$$

and

$$\frac{\delta G^{(i)}}{\sigma_G} = -\lambda_{Gq}\frac{\delta q_0^{(i)}}{\sigma_q} - \lambda_{G\alpha}\frac{\delta\alpha^{(i)}}{\sigma_\alpha} + \nu_G^{(i)} \tag{A6}$$

Using $G = \alpha\tau$ and $\alpha = \langle\alpha\rangle + \delta\alpha$, we can write

$$G = \langle\alpha\rangle\tau + \tau\delta\alpha = \langle\alpha\rangle\langle\tau\rangle + \langle\alpha\rangle\delta\tau + \langle\tau\rangle\delta_\alpha + \text{nonlinear terms} + \text{noise} \tag{A7}$$

And therefore we obtain the equivalence between the two parameterizations

$$\lambda_{Gq} = \langle\alpha\rangle \frac{\sigma_\tau}{\sigma_G} \lambda_{\tau q} \tag{A8}$$

and

$$\lambda_{G\alpha} = \langle\alpha\rangle \frac{\sigma_\tau}{\sigma_G} \lambda_{\tau\alpha} - \langle\tau\rangle \frac{\sigma_\alpha}{\sigma_G} \tag{A9}$$

In principle α can depend on size as well

$$\alpha = \langle\alpha\rangle - \sigma_\alpha \lambda_{\alpha q} \frac{\delta q}{\sigma_q} + \nu_\alpha \tag{A10}$$

B. Measuring the coupling coefficients λ_{XY}s from correlations

The parameter $\lambda_{\alpha q}$ can be estimated as

$$\lambda_{\alpha q} = -\frac{\mathrm{cov}(\alpha, q)}{\sigma_q \sigma_\alpha} = -\mathrm{cor}(\alpha q) := c_{\alpha q} \ . \tag{A11}$$

Analogously, λ_{Gq} and $\lambda_{G\alpha}$ can be obtained from correlation between variables. Since by definition

$$G = \langle G\rangle - \sigma_G \lambda_{Gq} \frac{\delta_q}{\sigma_q} - \sigma_G \lambda_{G\alpha} \frac{\delta_\alpha}{\sigma_\alpha} + \nu_G \tag{A12}$$

We then have

$$\mathrm{cov}(G, q) := \langle \delta q G\rangle = -\sigma_G \sigma_q \lambda_{Gq} - \sigma_G \lambda_{G\alpha} \frac{\langle \delta_\alpha \delta q\rangle}{\sigma_\alpha} = -\sigma_G \sigma_q \lambda_{Gq} - \sigma_G \lambda_{G\alpha} \lambda_{\alpha q} \sigma_q = -\sigma_G \sigma_q \left(\lambda_{Gq} + \lambda_{G\alpha} \lambda_{\alpha q}\right) \ , \tag{A13}$$

and therefore we have

$$c_{Gq} := \frac{\langle \delta q G\rangle}{\sigma_G \sigma_q} = -\lambda_{Gq} - \lambda_{G\alpha} \lambda_{\alpha q} \ . \tag{A14}$$

Similarly, we obtain

$$\langle G \delta\alpha\rangle = -\sigma_G \lambda_{Gq} \frac{\langle \delta\alpha \delta q\rangle}{\sigma_q} - \sigma_G \lambda_{G\alpha} \sigma_\alpha = \sigma_G \sigma_\alpha \left(-\lambda_{Gq} \lambda_{\alpha q} - \lambda_{G\alpha}\right) \ , \tag{A15}$$

which simplifies to

$$c_{G\alpha} = -\lambda_{Gq} \lambda_{\alpha q} - \lambda_{G\alpha} \ . \tag{A16}$$

Solving for $\lambda_{G\alpha}$ and λ_{Gq}, we obtain

$$\lambda_{Gq} = -\frac{c_{Gq} + c_{G\alpha}c_{\alpha q}}{1 - c_{\alpha q}^2} \ , \tag{A17}$$

and

$$\lambda_{G\alpha} = -\frac{c_{G\alpha} + c_{Gq}c_{\alpha q}}{1 - c_{\alpha q}^2} \ , \tag{A18}$$

In a similar way we can estimate $\lambda_{\tau q}$ and $\lambda_{\tau\alpha}$ from equation A5, obtaining

$$\lambda_{\tau q} = -\frac{c_{\tau q} + c_{\tau\alpha}c_{\alpha q}}{1 - c_{\alpha q}^2} \ , \tag{A19}$$

and

$$\lambda_{\tau\alpha} = -\frac{c_{\tau\alpha} + c_{\tau q}c_{\alpha q}}{1 - c_{\alpha q}^2} \ , \tag{A20}$$

Note that $G^{(i)} - \langle G\rangle = \delta q^{(i+1)} - \delta q^{(i)}$. We have therefore

$$\sigma_G^2 = \langle\left(\delta q^{(i+1)} - \delta q^{(i)}\right)^2\rangle = 2\sigma_q^2 - 2\langle\delta q^{(i+1)}\delta q^{(i)}\rangle = 2\sigma_q^2 - 2\langle\left(\delta G^{(i)} + \delta q^{(i)}\right)\delta q^{(i)}\rangle = -2\langle G^{(i)}\delta q^{(i)}\rangle \ . \tag{A21}$$

We have therefore

$$\sigma_G^2 = 2\sigma_G\sigma_q\left(\lambda_{Gq} + \lambda_{G\alpha}\lambda_{\alpha q}\right) \ , \tag{A22}$$

and then

$$\sigma_G = 2\sigma_q\left(\lambda_{Gq} + \lambda_{G\alpha}\lambda_{\alpha q}\right) \ , \tag{A23}$$

C. Mechanistic interpretation of $\lambda_{\alpha q}$: correlation of α across generations

The growth rate α is correlated across generations. A minimal model to express this correlations is to consider the following dynamics for the growth rate $\alpha^{(i+1)}$ at generation $i+1$

$$\alpha^{(i+1)} = (1-\rho)\langle\alpha\rangle + \rho\alpha^{(i)} + \nu_\alpha^{(i)} = \langle\alpha\rangle + \rho\delta\alpha^{(i)} + \nu_\alpha^{(i)} \ . \tag{A24}$$

It is simple to show that, at stationarity,

$$\langle\delta\alpha^{(i+1)}\delta\alpha^{(i)}\rangle = \rho\langle(\delta\alpha^{(i)})^2\rangle = \rho\sigma_\alpha^2 \ , \tag{A25}$$

and therefore ρ is the correlation of the mother and the daughter growth rate.

Using the definition of G we have that

$$\delta q^{(i+1)} - \delta q^{(i)} = q_0^{(i+1)} - q_0^{(i)} = G^{(i)} - \langle G\rangle \ , \tag{A26}$$

and therefore

$$\delta q^{(i+1)} = (1 - \frac{\sigma_G}{\sigma_q}\lambda_{Gq})\delta q^{(i)} + \sigma_G\lambda_{G\alpha}\frac{\delta\alpha^{(i)}}{\sigma_\alpha} + \nu_G^{(i)} \ . \tag{A27}$$

From equation A24, we obtain

$$\langle\delta\alpha^{(i+1)}\delta q^{(i+1)}\rangle = \rho\langle\delta\alpha^{(i)}\delta q^{(i+1)}\rangle \ . \tag{A28}$$

From equation A27 we have

$$\langle\delta\alpha^{(i)}\delta q^{(i+1)}\rangle = (1 - \frac{\sigma_G}{\sigma_q}\lambda_{Gq})\langle\delta\alpha^{(i)}\delta q^{(i)}\rangle + \sigma_G\lambda_{G\alpha}\frac{\langle(\delta\alpha^{(i)})^2\rangle}{\sigma_\alpha} \ . \tag{A29}$$

At stationarity $\langle\delta\alpha^{(i)}\delta q^{(i)}\rangle = \langle\delta\alpha^{(i+1)}\delta q^{(i+1)}\rangle = \langle\delta\alpha\delta q\rangle$ and $\langle(\delta\alpha^{(i)})^2\rangle = \sigma_\alpha^2$. Combining equation A28 and equation A29, we have

$$\frac{1}{\rho}\langle\delta\alpha\delta q\rangle = (1 - \frac{\sigma_G}{\sigma_q}\lambda_{Gq})\langle\delta\alpha\delta q\rangle + \sigma_G\lambda_{G\alpha}\sigma_\alpha \ , \tag{A30}$$

and therefore

$$\lambda_{\alpha q} = \frac{\langle\delta\alpha\delta q\rangle}{\sigma_\alpha\sigma_q} = \frac{\sigma_G}{\sigma_q}\frac{\rho\lambda_{G\alpha}}{1 - \rho^2(1 - \frac{\sigma_G}{\sigma_q}\lambda_{Gq})} \ . \tag{A31}$$

By introducing equation A23, we obtain

$$\lambda_{\alpha q} = 2\left(\lambda_{Gq} - \lambda_{G\alpha}\lambda_{\alpha q}\right)\frac{\rho\lambda_{G\alpha}}{1 - \rho(1 - 2\left(\lambda_{Gq} - \lambda_{G\alpha}\lambda_{\alpha q}\right)\lambda_{Gq})} \ . \tag{A32}$$

By solving this equation for $\lambda_{\alpha q}$ we get

$$\lambda_{\alpha q} = \frac{-\left(1 - \rho + 2\rho(\lambda_{Gq}^2 - \lambda_{G\alpha}^2)\right) \pm \sqrt{16\lambda_{G\alpha}^2\lambda_{Gq}^2\rho^2 + \left(1 - \rho + 2\rho(\lambda_{Gq}^2 - \lambda_{G\alpha}^2)\right)^2}}{4\lambda_{G\alpha}\lambda_{Gq}\rho} \tag{A33}$$

D. Mechanistic interpretation of $\lambda_{G\alpha}$: adder with fluctuating growth rate

As explained in the text, we consider two alternative ways to include fluctuations of growth rate the adder model. In the second part of this section we generalize it to a general control mechanism.

In the first scenario, the size at division is given by

$$V_f = (V_0 + \Delta(\langle\alpha\rangle))\, e^{\nu} \ , \tag{A34}$$

where the added size Δ is a function of the average growth rate only, and ν is a noise term. In the second scenario, we assume

$$V_f = (V_0 + \Delta(\alpha))\, e^{\nu} \ , \tag{A35}$$

where Δ is a function of the individual growth rate.

The Shaecter's law can be written as

$$\langle \log \Delta \rangle = \log S_0 + \langle\alpha\rangle T \ , \tag{A36}$$

and implies that, under both scenarios,

$$\Delta(a) = S_0 \exp(aT) \ . \tag{A37}$$

The adder equations can be written as

$$q_f - q_0 = \log\left(1 + \frac{\Delta}{V_0}\right) + \nu = \log\left(1 + \exp(\log\Delta - \log V_0)\right) + \nu \ . \tag{A38}$$

Under the first scenario, Δ is constant within a condition, and we can rewrite

$$q_f - q_0 = \log\left(1 + \exp(\langle\log\Delta\rangle - \langle q_0\rangle - \delta q_0)\right) + \nu \ . \tag{A39}$$

By expanding this equation around the average, we obtain $\langle \log\Delta\rangle = \langle q_0\rangle$ and

$$G = q_f - q_0 = -\frac{1}{2}\delta q_0 + \nu \ , \tag{A40}$$

which implies $\lambda_{Gq} = 1/2\sigma_q/\sigma G$ and $\lambda_{G\alpha} = 0$.

Under the second scenario, Δ depends on growth rates fluctuations, and we obtain

$$q_f - q_0 = \log\left(1 + \exp(\langle\log\Delta\rangle - \langle q_0\rangle + T\delta\alpha - \delta q_0)\right) + \nu \ . \tag{A41}$$

By expanding this equation around the average, we obtain again $\langle \log\Delta\rangle = \langle q_0\rangle$ and

$$G = q_f - q_0 = -\frac{1}{2}\delta q_0 + \frac{T}{2}\delta + \nu \ , \tag{A42}$$

which implies $\lambda_{Gq} = 1/2\sigma_q/\sigma G$ and

$$\lambda_{G\alpha} = \frac{\sigma_\alpha}{\sigma G}\frac{T}{2} \ . \tag{A43}$$

This argument can be generalized to an arbitrary mechanism of size control (11)

$$G = q_f - q_0 = g\left(\frac{V_0}{V^*}\right) + \nu \ , \tag{A44}$$

where V^* is the only dimensional scale of the process, and $g(1) = \log 2$. Note that the existence of a unique dimensional scale is a necessary and sufficient condition for the collapse of the size distribution. The other assumption is that V^* follows the Shaecter's law. It is simple to see that in the first scenario, when V^* depends only on the average growth rate, $\lambda_{G\alpha} = 0$. In the second scenario (where we assume $V^* = S_0 e^{\alpha T}$), we can expand V_0 and V^* around the average to obtain

$$G = q_f - q_0 = g_1 \delta q_0 - g_1 T \delta\alpha + \nu \ , \tag{A45}$$

where $g_1 = g'(1)$. We have therefore that $\lambda_{G\alpha} = \frac{\sigma_\alpha}{\sigma G} g_1 T$. Since by definition $g_1 = -\lambda_{Gq}\sigma_G/\sigma q$, we obtain

$$\lambda_{G\alpha} = -\frac{\sigma_\alpha}{\sigma q}\lambda_{Gq} T \ . \tag{A46}$$
